# Discovering the Use of Complementary and Alternative Medicine in Oncology Patients: A Systematic Literature Review

**DOI:** 10.1155/2021/6619243

**Published:** 2021-01-13

**Authors:** Fatmah Alsharif

**Affiliations:** Medical Surgical Nursing Department, School of Nursing, King Abdulaziz University, Jeddah, Saudi Arabia

## Abstract

**Background:**

Patients with cancer pursue all possible opportunities of effective remedies. In Saudi Arabia, most patients have tried complementary medicine during their cancer treatment plan; however, some complementary medicines might pose a danger to health. In Arab countries, studies regarding the use of complementary medicines and the intentions behind using complementary medicines among cancer patients are inadequate and all but nonexistent. The aim of this review was to focus on demographic, prevalence, and reasons for complementary and alternative medicine use among patients with cancer.

**Methods:**

A rigorous literature search was conducted for articles published in the English language, using the search terms “complementary and alternative medicine,” “oncology,” “malignancy” AND “cancer patients” in five scientific databases, namely, Medical Literature On-Line (MEDLINE), PubMed, Web of Science, Psychology Information (PsycINFO), and Google Scholar, from 2010 to 2020.

**Results:**

Of the 13,160 studies returned from the search, only 12 were eligible and included in this review. The combined prevalence for using complementary and alternative medicines across all studies totaled 25%–80% of cancer patients for the treatment of their cancers. Natural products, including vitamins and minerals, herbal products, and relaxation, were the most common type of complementary and alternative medicines used. The reason for using complementary and alternative medicines was reported to be their helpfulness in recovering, healing, and improving health. Most of the studies mentioned that participants obtained their complementary and alternative medicines from multiple sources, including the media, family and friends, and physicians.

**Conclusion:**

The use of complementary and alternative medicines in cancer patients can be inferred as an attempt to investigate all possibilities, a manifestation of a coping style, or an illustration of unmet desires in the cancer management continuum. Anyhow, there should be adequate communication between healthcare providers and patients, which is crucial for establishing a trusting healthcare provider-patient relationship. *Relevance to clinical practice*. It is crucial that healthcare providers explore the use of complementary and alternative medicines with their cancer patients, as well as educating them about the possible usefulness of therapies based on the available evidence.

## 1. Introduction

Cancer is deliberated as a common health problem, associated with significant disabilities, and is one of the three leading causes of death worldwide [1]. According to the World Health Organization (WHO), it is estimated that cancer rates will be increased by 50% to 15 million by 2030. Thus, most cancer patients and their families seek all possible options related to effective treatment to manage the trauma of distress, worries, and immense fear associated with their condition [2, 3].

Some patients explore complementary and alternative medicine (CAM) choices, which constitute a type of medicine independent of conventional medicine. This practice is widely used across the world and can be explained as any usage that is not part of conventional medicine [4]. It is commonly the application of the sum of knowledge and practices developed through experiences and beliefs of distinctive cultures [1].

Numerous patients that feel worried decide to leave their course of conventional medicine and instead seek help via complementary and alternative medicines, which are therefore gaining increasing interest, especially in the field of oncology [5]. Due to this trend, patients' interests should be given due deliberation in current oncology practices and should be addressed in such a way as to provide recommendations regarding acceptable and individualized therapy merged with supportive care, including CAMs, under standardized oncology protocols [6]. However, the data pertaining to CAMs and their inappropriate management are a challenge; for example, herbal supplies, which are considered a complementary medicine, may interact with conventional treatments, potentially exposing the patient to an increased risk of ill health. Moreover, CAMs are potentially subject to corruption, contamination, or replacement with other more harmful products [7]. The literature of this study aimed to summarize the use of CAMs among patients with cancer. This review involved the following main phases: data search strategy, article selection, data extraction, data analysis/synthesis, and critical appraisal of the included articles [8]. ‬ The particular questions about patients with cancer were as follows [2]: Which types of CAM were stated [9]? What were the sources of information about CAM [4]? What factors contributed to CAM use? [7] Why did patients decide to use CAM?

## 2. Methodology

Systematic reviews promote a present basis for any observation and might broaden new study ideas. At the beginning of a written document, it allows readers to enjoy and become expertise in the contemporary tendencies of troubles and clears up the importance of revolutionary studies [10]. Tappen (2016) demonstrated that literature reviews involve methodically determining, finding, and examining materials that are associated with the study question [8]. The literature of this study aimed to discuss the use of CAMs among patients with cancer. This review involved the following main phases: data search strategy, article selection, data extraction, data analysis/synthesis, and critical appraisal of the included articles [8]. ‬

### 2.1. Data Search Strategy

This study was carried out by analyzing the literature through PRISMA's evidence-based data evaluation search strategy. It illustrates the reporting of randomized study assessment evaluations, although it can also be used as a framework for reporting systematic reviews of other types of research.

In order to extract the data for the factors highlighted, through the systematic use of electronic databases, the investigator collected the appropriate published papers and articles. Nonetheless, a number of the foremost inclusive journal places that used to cognizance the literature in the field of CAM research are Medical Literature On-Line (MEDLINE), PubMed, Web of Science, Psychology Information (PsycINFO), and Google Scholarreplace.

The following keywords were applied for the electronic search: “Complementary medicine,” “alternative medicine,” “oncology,” “malignancy,” “cancer,” and “cancer patients.” These keywords were used in combination or separately. Furthermore, the reference sections of the relevant articles were checked to distinguish additional trials unexploited by the electronic search. There were some digital databases that confirmed obsolete in attempting to find the associated articles via keywordsreplace.

The selected electronic records were chosen on the basis of the extensive range of disciplines they covered and their integrity, in addition to those almost certainly about issues relevant to Saudi Arabia.

### 2.2. Article Selection and Data Extraction

The articles were selected based on predefined inclusion and exclusion criteria. The research articles and journals were examined critically through and extracted from online computerized search engines. The inclusion criteria for the research articles were as follows:

1. Published in English language only
2. A systematic search of peer-reviewed, published literature from 2010 to 2020 conducted between March and August 2020
3. Focusing on the CAMs used by cancer patients
4. Relying on both qualitative and quantitative evidence or on mixed research methodsreplace
5. Including a list of references of all reviewed articles with an appropriate inclusion quality, and with no follow-up by the investigators to extract further primary or secondary data

Meanwhile, articles were excluded if

1. they were unpublished articles or studies
2. they were opinions or commentaries
3. they were not published in the English language

Primarily, the titles and abstracts of relevant articles recognized during the electronic database search were scrutinized and included or excluded based on the predefined selection criteria listed above. The population, intervention, comparison, outcomes, and study design parameters were used to describe the eligibility criteria.

The data pertaining to the objectives, sample population, design, methodology, and data collection procedures were extracted from the selected articles. In addition, findings, discussions, and conclusions were also analyzed in order to verify the relationship among some of the variables. The variables that were searched were those related to CAM use in cancer patients. All the data collected are clustered, summarized and compared for analysis in the studies.

### 2.3. Data Analysis and Synthesis

In order to thoroughly reflect on the literature reviews, the research reviewed andanalysis several relevant studies within the 2010–2020 period. Some of the studies highlighted and addressed Some common problems that countries might have come to know during catastrophic situations in which an extensive number of fully competent nurses would have been required. In order to demonstrate these points, the researchers made significant use of all current journal articles, reports, editorials, and correlational descriptive studies. In order to better understand the point of view of nurses on their lack of expertise in delivering good services, the study also provided a keen emphasis on both primary and secondary research papers.

### 2.4. Critical Appraisal of the Included Studies

The included studies were critically appraised; they were evaluated for their psychometric measures such as reliability and validity, the two necessary features that determine the quality of a quantitative study. The reliability of quantitative research is correlated with its consistency, stability, agreement, reproducibility, repeatability, and homogeneity. Nevertheless, validity refers to how well-founded and accurate an instrument or a study measure is [10].

The citations used in this research paper have been correctly cited and cover the key topic-related primary headings. Approximately 600 articles and papers that were not in accordance with the study purpose were also omitted by the investigator.

## 3. Results

**Figure 1:**
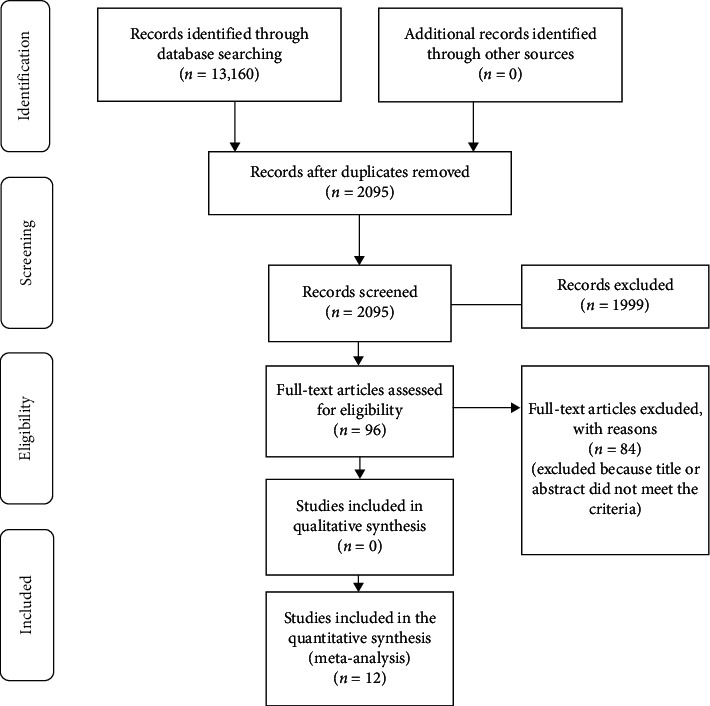
Flow diagram of the different phases of the systematic review.

‬From the database, 13,160 articles (2010–2020) were identified, of which 12 met the criteria for inclusion. The documents were original quantitative research articles, published in English and local languages, and directly relevant to the aim of the study (Table 1). Four of the studies (33.3%) were conducted in Asia, followed by three studies (25%) in Arab countries, two studies (16.7%) in Canada, two (16.7%) in Europe, and one (8.3%) in South America (Figure 1).

### 3.1. Sociodemographic Factors

The sociodemographic factors found to be correlated with the practice of CAM enclosed age, education level, income, marital status, and presence of a support group. Most of the samples in the studies were females, aged over 40 years and married, and had moderate level of income [1, 2, 4, 11–13]. Other studies reported on middle-aged males with a high income (Table 1) [9, 14].

Out of the 12 studies that investigated sociodemographic factors and the use of CAMs in cancer patients, six reported that younger female patients with a moderate education level were more likely to use CAMs than those who were male, older, and had a lower education level [1, 2, 9, 12, 13, 15]. Three studies reported that males were more likely to use CAMs than females [5, 11, 14]. Only a few studies found that age and education were not related to the use of CAMs in cancer patients [4, 7, 16].

Of the 12 studies, eight reported that cancer patients who had a higher income were more likely to use CAMs than those who had a lower income [1, 7, 9, 12, 16]. Meanwhile, two studies reported that those with low income were more likely to use CAMs than those who had a higher income [2, 4, 15], and other study found a similar relationship between low and moderate incomes (rather than a high income) [13]. However, only one study reported a lack of a relationship between income and CAM use [11].

Regarding marital status, seven studies revealed that married patients were more likely to use CAMs than those who were unmarried [1, 2, 7, 9, 12, 13, 16]; however, three studies showed no relationship between marital status and CAM use (Table 1) [5, 11, 14].

### 3.2. Types of Complementary and Alternative Medicines Used

Five studies mentioned that natural products, including minerals, vitamins, and relaxation, were the common types of CAMs used [1, 2, 4, 7, 16]. Four studies reported that unlabeled fresh and processed herbal products and honey were the most commonly used complementary medicines. Porcupine flower, lingzhi, *Ephedra foeminea* (Alanda) [2, 4, 11, 14], Chinese medicine, reflexology, and hypnosis were also reported to be common [5]. One study mentioned that cancer patients also used traditional medicines [15]. Two studies conducted in Arab countries (Tunisia and Egypt) mentioned that cancer patients' use of CAMs was religious-based [2, 11], while in a study conducted in Saudi Arabia, most of the cancer patients used Quran recitation, supplication, Zamzam water, olive oil, and black seeds (*Nigella sativa*) [9].

Among the reports, the reported incidence of use of each item varied. In general, the most prevalent natural commodity in use was natural merchandise containing various plant parts prepared in various ways. Only two of the included studies did not report the forms of CAMs used by their study participants (Table 1) [12].

### 3.3. Reasons for Using Complementary and Alternative Medicines

The reasons for using of CAMs reported by cancer patients were diverse, and some reported more than one reason. All 12 studies reported that patients used complementary and alternative medicines because they believed it was helpful to their recovering and healing and improved their health [1, 2, 4, 5, 7, 9, 11–16].

The other reasons included the enhancement of physical and emotional well-being, as well as rising their body's capability to fight cancer [1, 2, 5, 9, 14], strengthening their immune system [4, 5, 9, 12], following their physician's suggestions [4], controlling their pain, and improving their appetite [9, 15]. CAMs were predominantly used to prevent or treat the side effects of anticancer treatments [5, 15]. One study did not mention any specific reasons, but the participants said they were just trying to do everything that could help them [16].

Additionally, the results of some of the studies showed that the overall degree of satisfaction regarding complementary and alternative medicine usage was generally high [5, 9, 11, 15], while one study showed low satisfaction of the use of complementary and alternative medicines [13].

### 3.4. Source of Information

Most of the studies mentioned that patients obtained information about CAMs from multiple sources. Internet (media) and the social network Facebook were the main sources of information regarding CAMs, followed by family and friends [7, 9, 11, 14, 16]. The most reliable source of information about complementary medicines was reported to be physicians [4, 5, 15]. Three studies reported that cancer patients discussed their CAM use with cancer care providers/physicians and nurses [5, 9, 11, 13, 15].

## 4. Discussion

Herein, it was found that approximately 25%–80% of patients with cancer in the included 12 studies testified to the use of CAMs for the management of their cancer. The high prevalence of CAM use has been reported in recently published studies on CAM use among patients with different kinds of cancers [17, 18].

In this systematic review, the researcher found that patients with cancer practiced different types of CAMs, such as natural products, dietary supplements, prayers, and vitamins [1, 2, 4, 7, 16]. However, none of the studies mentioned the patients' perceptions regarding how well the CAMs they used worked in terms of improving their health. However, a few of the studies assessed patients' satisfaction of using CAMs [5, 9, 11, 13, 15].

Most of the patients did not discuss the use of CAMs to their healthcare providers [1, 9, 11–15]. The motivation for nondisclosure covered fear of a terrible reaction by their healthcare specialists, not being addressed regarding CAMs, and the perceived low knowledge of healthcare providers on CAMs, and thus there being no necessity to explore the topic with them [17, 18].

Patients, regardless of the type of cancer they had, practiced and used CAMs for numerous reasons and held various expectations, consisting of a cure for their cancer, control of most of their cancer-related symptoms, improving their immune system, and enhancing their physical and psychological well-being. The use of CAMs is hence likely to endure alongside standardized conventional cancer management and treatment, primarily because it has long been part of the culture of individuals and patients may thus trust CAM providers, and because of the convenient strategies and expense of CAMs [4, 5, 13, 15]. However, some other studies have reported that patients do not believe complementary and alternative medicines have an anticancer effect [12, 14].

Some of the other reviewed studies also showed that patients who use CAMs do so due to their dissatisfaction of conventional treatments, to the numerous side effects of standardized cancer drugs, and to their fear of surgery experiences, in addition to the fact that CAMs are more easily accessible and are less expensive than conventional treatments [2, 5, 7, 9, 11, 16].

## 5. Conclusions

The majority of patients with cancer in the reviewed studies used various different types of CAMs concurrently with their cancer treatment. The use of CAMs in cancer patients can be inferred as an attempt to discover all potential options, an illustration of a coping style, or unmet desires in their current cancer care trajectory. In every cancer case, there should be adequate communication between patients and their healthcare providers, which is crucial for establishing physician–patient relationship trust.

## Figures and Tables

**Table 1:** Studies related to using complementary and alternative medicine.

	Author, year, and location of the study	Study design	Participants	Aim of study	Type of complementary and alternative medicine	Instrument/tool	Result	Outcome measures
1	Wode, K., Henriksson, R., Sharp, L., Stoltenberg, A., and Nordberg, J.H. (2019) Sweden.	Cross-sectional.	Cancer patients.	To explore Swedish cancer patients´ patterns of CAM use, their experiences, and preferences.	Natural products including vitamins and minerals and relaxation, mind and body practices, changes in diet, anthroposophic medicine, homeopathy, energy medicine, traditional Chinese medicine.	Questionnaire.	Lifetime CAM use was reported by 34% (*n* = 256), and 26% (*n* = 198) used CAM after cancer diagnosis. Being female, younger, and having higher education predicted CAM use. Most commonly used methods were natural products. Main reasons for CAM use were improvement of physical, general, and emotional well-being and increasing the body's ability to fight cancer. Satisfaction with CAM usage was generally high. Reported adverse effects were few and mild; 54% of users spent < 50 euros a month on CAM. One third had discussed their CAM use with cancer care providers. More than half of all participants thought that cancer care providers should be able to discuss (58%) and to consider (54%) the use of CAM modalities in cancer care.	CAM use and related experiences among Swedish patients with solid tumors in both curative and palliative stage.
2	Buckner, C.A., R M lafrenie, Dénommée, J.A., Caswell, J.M., and Want, D.A. (2018) Ontario.	A cross-sectional.	141 cancer patients.	To determine the rate of cam use in patients receiving treatment at a Northern Ontario cancer centre.	Biologic products, vitamins, and minerals.	Anonymous questionnaire.	Patients in Northern Ontario reported significant cam use both before and after diagnosis. However, as a function of the cam type, cam use was greatly enhanced after cancer diagnosis. For example, the number of patients who reported use of biologic products increased to 51.8% after a cancer diagnosis from 15.6% before a cancer diagnosis. Patients reported much smaller changes in the use of alternative medical systems or spiritual therapy after diagnosis. Vitamin use was reported by 66% of respondents, and the number of different cams used correlated significantly with the reported number of vitamins used.	Rate of cam use in patients receiving treatment at a Northern Ontario cancer centre.
3	Balneaves, Wong, Porcino, Truant, Thorne, and Wong, (2018) Canada.	A sequential, multimethod research design.	Chinese-speaking cancer patients.	To explore Chinese-speaking cancer patients (CSCPs) use of complementary and alternative medicine (CAM), sources of CAM information, and decision support needs, as well as their experience of making CAM decisions.	Biologically based therapies, energy-based therapies, body-based therapies, and mind-body therapies.	Semistructured interviews.	More than 65% of CSCPs reported using CAM. CSCPs favored biologically based therapies, including traditional Chinese medicine herbs and other natural health products. Many CSCPs were using CAM without adequate culturally appropriate information and decision support. Those who made decisions spontaneously relied on peers for advice whereas deliberate decision makers sought information from multiple sources, including peers and the Internet, selecting therapies congruent with their cultural health perspectives, and previous experiences with CAM.	Use of complementary and alternative medicine (CAM) experience of making CAM decisions.
4	Gras, M., et al. (2019). France.	A prospective survey.	200 cancer patients.	To report on the nature, frequency of use, and patient-reported outcome of CAMs in a single-center study.	Osteopathic medicine, homeopathy, acupuncture, healing touch, magnetism, naturopathy, suction cups, Chinese medicine, reflexology, to hypnosis.	A questionnaire.	CAMs ranged from osteopathic medicine, homeopathy, acupuncture, healing touch, magnetism, naturopathy, suction cups, Chinese medicine, reflexology, to hypnosis. CAMs were widely used (*n* = 166, 83%), the first being osteopathic medicine (*n* = 99, 49.5%), the second homeopathy (*n* = 78, 39.0%), and finally acupuncture (*n* = 76, 38.0%). Whatever the CAM, high satisfaction rates were reported (median satisfaction: 61–81%). CAMs were mainly used to prevent/treat side effects of anticancer treatments (81.2% for healing touch), increase well-being (55.4% for naturopathy), improve the immune system (16.9% for homeopathy), and treat cancer (*n* = 3, 5.1% for homeopathy). Patients could easily consider using CAMs, as up to 50.8% would have accepted a consultation.	Use of CAMs and level of satisfaction.
5	Amirmoezi, F., et al. (2017). Iran.	A cross-sectional study.	36 cancer patients.	To determine the use of CAM and associated factors among Iranian cancer patients, reasons behind this use, satisfaction, and information about complementary and alternative therapy.	Minerals and vitamins, herbals, and miscellaneous health practice.	A questionnaire.	A total number of 36 cancer patients participated in this study. The majority of respondents (94.4%) used or were using at least one CAM product or practice till the study date. Altogether, the most common type of CAM used among cancer patients was praying (86.1%). The most popular herbs in herbals subgroup were mint and garlic with 41.7%. Multivitamins and vitamin C were more favored than other vitamins and minerals (33.3%). No major side effects were reported regarding CAM use. The most reliable source of information about complementary medicine was reported to be physicians.	
6	Abdelmoaty, A.M., et al. (2018). Egypt.	A cross-sectional study.	331 cancer patients.	Assessing the pattern of use, motives, and possible predictors of complementary cancer therapy among a sample of Egyptian oncology patients.	Honey and herbal medicine were the most commonly used CM.	Reviewing of the patient medical records, and personal interviews.	Of the included patients, 76 out of 331 (about 23%) used CM during their cancer treatment course; none of them used any alternative cancer therapies. Being a female patient with low educational attainment (secondary or less) was significantly associated with the use of CM (OR = 1.78, *P*=0.018 and OR = 2.90, *P*=0.011, resp.). The dominated reason for using CM therapy was to increase their body's ability to fight cancer (31.6%). Dissatisfaction with therapy was a significant positive predictor for CM usage (OR = 2.10, *P*=0.006). Honey and herbal medicine were the most commonly used CM. More than 60% of the patients who used CM (48/76) did not inform their treating physicians about CM usage and almost 23% of (11/48) responded that the physician would disapprove it.	Use, motives, and possible predictors of complementary cancer therapy among Egyptian oncology patients.
7	Labidi, S., Ennouri, S., Rachdi, H., El Benna, H., Mejri, N., Daoud, N., Berrazaga, Y., and Boussen, H. (2020). Tunisia.	A cross-sectional survey.	120 adult patients.	To explore the use of complementary and alternative medicine (CAM) and to identify their side effects, when used in cancer patients.	Wild herbs, *Ephedra foeminea* (Alanda).	An anonymous questionnaire to assess complementary and alternative medicine use.	One hundred twenty patients participated in the survey; among them, 102 used CAM (85%). A majority of users were female patients (*n* = 72, 70.6%), and mean age was 52.4 years ± 11.6. Patients had breast cancer in 48% of cases. Wild herbs were the most commonly used alternative therapy (67.7%), particularly *Ephedra foeminea* (Alanda) in 52% of cases. Patients' families incited them to use CAM in 64.7% of cases. Internet and social network (Facebook) were the major sources of information on CAM (79.4%), followed by family and friends (72.5%). Fourteen patients (13.7%) reported nausea and vomiting secondary to CAM use. We reported disruption of liver function in 9.8% of cases and renal failure in 1.96%, with fatal issue in one patient using *Ephedra*. Nineteen patients (18.6%) informed their oncologist about the alternative therapy they received.	Use of complementary and alternative medicine (CAM) and to identify their side effects, when used in cancer patients.
8	Chotipanich, A., sooksrisawat, C., and jittiworapan, B. (2019).	A cross-sectional study design.	426 patients with various cancers.	To investigate the patterns of complementary and alternative medicine use and its association with time to conventional treatment.	Unlabeled fresh and processed herbal products, porcupine flower, lingzhi mushroom, commercial fruit and vegetable extract beverage, Aloe vera juice, and fish oil.	A semistructured interview protocol.	The results indicated that 192 of the 426 patients (45.1%) reported using complementary and alternative medicines; herbal products were the most common type. Approximately 34.3% of these medicines involved unlabeled herbal products with unidentifiable components. The rates of complementary and alternative medicine use were significantly elevated for men and patients with stage IV cancer. The multivariable linear regression analysis of the relationship between factors and the time until conventional treatment was received revealed that the regression coefficient of the use of complementary and alternative medicine was 56.3 (95% confidence interval [27.9–84.6]). This coefficient reflected an additional 56.3 days of time until conventional treatment, relative to patients who did not use complementary and alternative medicine.	Rate of complementary and alternative medicine use.
9	Shetty, N., Rai, P.R., and Shetty, A. (2019). India.	Observational study.	407 patients.	To determine the prevalence of the use of traditional, complementary, and alternative medicine (CAM) by cancer patients visiting a cancer care centre. This study laid an emphasis on the predictors of the use of CAM.	Traditional medicine, allopathic medicine.	A questionnaire.	The prevalence of traditional medicine and CAM was found to be 23.5% (96 patients). The mean duration of CAM use was 4.8 months (0.25 months–48 months). About 77% of the users had an education level below the upper primary level, of which 30.02% were illiterate. About 62.5% of the users were below poverty line. Nearly, 41.7% of the patients had not received any allopathic treatment before starting traditional medicine and CAM and did so for a mean duration of 4 months. About 53% of the patients who received some form of traditional medicine and CAM claim to have experienced some symptomatic benefits from its use. Nearly, 68.75% of the users were simultaneously receiving conventional anticancer therapy. Traditional medicine and CAM use was disclosed to the treating physician by 55% of the patients.	Prevalence of the use of traditional medicine, complementary and alternative medicine (CAM) by cancer patients visiting a cancer care centre. This study laid an emphasis on the predictors of the use of CAM.
10	Shin. J., Kim S., Park, B., Park, J., Choi, J. Seo, H.G. (2012). Korea.	A cross-sectional study design.	2,661 cancer patients.	To explore factors predicting CAM use among a nationally representative sample of cancer patients.	-	Administered questionnaires.	Overall, 25.5% reported that they had used or were using CAM. Higher income, presence of metastasis, longer time since diagnosis, less trust in hospitals, lower overall satisfaction, and higher degree of informational need were significantly associated with CAM use.	Factors predicting CAM use among a nationally representative sample of cancer patients.
11	Ricardo, E.D., Oishi, D., Dos Santos, M.O., and D'Alpino, R.D. (2020). Brazil.	A cross-sectional survey.	156 patients.	To define CAM use by cancer patients and investigate factors that might influence it.	-	A questionnaire.	Most cancer types were breast (17.4%), colorectal (16.7%), and lung (16.1%) cancer. More than 90% of the participants were on any active treatment. The prevalence of current CAM use was 29.6%. 58.7% of the patients did not believe CAM has anticancer properties, including 32.6% of patients who reported CAM use. Two-thirds of the participants have never discussed CAM with their oncologists. Only 5.1% of the respondents would abandon conventional cancer treatment in order to use just CAM. Among CAM users, 55% referred multiple therapies use. Of those therapies, spiritual surgery was the most prevalent one. There was a significant higher proportion of females reporting CAM use (*p*=0,029) as well as a higher proportion of CAM use among younger patients (*p*=0,008).	Factors that might influence it.
12	Abuelgasim, K.A., Alsharhan, Y., Alenzi, T., Alhazzani, A., Ali, Y.Z., and Jazieh,A. (2018). Saudi Arabia.	A cross-sectional study.	156 patients.	To investigate the prevalence and pattern of CAM use by Saudi cancer patients. It also discusses the possible benefits and harm related to CAM use by cancer patients, and it explores the beliefs patients hold and their transparency with health care providers regarding their CAM use.	Supplication, Quran recitation, Zamzam water, water read upon Quran, olive oil, black seed (*Nigella sativa*), garlic, camel milk, honey, camel urine, known herbal remedies, multivitamins, and unknown herbal mixture.	Questionnaire.	The prevalence of CAM use was 69.9%; the most prominent types of CAM were those of a religious nature, such as supplication (95.4%), Quran recitation (88.1%), consuming Zamzam water (84.4%), and water upon which the Quran has been read (63.3%). Drinking camel milk was reported by 24.1% of CAM users, whereas camel urine was consumed by 15.7%. A variety of reasons were given for CAM use: 75% reported that they were using CAM to treat cancer, enhance mood (18.3%), control pain (11.9%), enhance the immune system (11%), increase physical fitness (6.4%), and improve appetite (4.6%). 30% of CAM users had discussed the issue with their doctors; only 7.7% had done so with their nurses.	Prevalence and pattern of CAM use by Saudi cancer patients. It also discusses the possible benefits and harm related to CAM use by cancer patients, and it explores the beliefs patients hold and their transparency with health care providers regarding their CAM use.

## Data Availability

No data were used to support this study.

## Abbreviations

CAM: Complementary and alternative medicines.
